# Diagnosis and Prognosis of Prostate Cancer from Circulating Matrix Metalloproteinases and Inhibitors

**DOI:** 10.1155/2018/7681039

**Published:** 2018-07-10

**Authors:** William Khalil El-Chaer, Clayton Franco Moraes, Otávio Toledo Nóbrega

**Affiliations:** ^1^University of Brasília (UnB), 70910-900 Brasília, DF, Brazil; ^2^Catholic University of Brasília (UCB-DF), 71966-700 Brasília, DF, Brazil

## Abstract

Although prostate cancer (PCa) is the sixth most common type of neoplasm in the world and the second in prevalence among men (10% of all cases), there is shortage of studies focused on primary prevention of the disorder as well as little understanding on its pathophysiology. Currently, the PCa screening tools are the prostate specific antigen (PSA) dosage conjugated to rectal examination and confirmed by prostate biopsy. Despite the name, the PSA presents reduced specificity, being necessary the identification of new biomarkers that allow an earlier and more precise diagnosis and even better prognosis. Several studies have associated matrix metalloproteinases (MMPs) and their tissue inhibitors (TIMPs) to PCa tumorigenesis and metastasis. Most of the studies so far have been carried out by investigating in situ expression of the metalloproteinases, either by transcriptional measures or by immunohistochemistry with biopsy or postoperative tissue. Investigations in human plasma and serum are scarce, and a bibliographical search resulted in 17 studies which are presented and interpreted herein. This narrative review discusses their settings and findings along with aspects related to circulating metalloproteinases as potential biomarkers for diagnosis or prognosis of the prostatic malignancy, expressing the authors' reticent view on their applicability due to the poor quality of evidence available.

## 1. Introduction

Statistics worldwide indicate that prostate cancer (PCa) has high prevalence and lethality, with three-quarters of cases among 65-year-oldsters [1]. Serum prostate specific antigen (PSA) levels are measured for early detection, staging, and monitoring despite not being a specific marker for PCa, rising in cases of prostatitis or benign prostatic hyperplasia (BPH) [2, 3].

Biochemical evidence indicates that serum proteinases, namely, matrix metalloproteinases (MMPs), play key roles in the pathophysiology of this malignancy. MMPs are zinc- or calcium-dependent endopeptidases that degrade various components of extracellular matrix, mainly collagen, elastin, laminin, fibronectin, and proteoglycans, being involved in tumorigenesis and metastasis to favor migration of tumor cells besides being proangiogenic [3].

Twenty-four MMPs have been identified, including collagenases (MMP-1, 8, 13, and 18), gelatinases (MMP-2 and 9), stromelysins (MMP-3 and 10), matrilisins (MMP-7 and 26), and membrane-type MMPs (MMP-14, 15, 16, 17, 24, and 25), among other types. They are found in all tissues and in plasma, being secreted mostly as pro-MMPs activated by the urokinase-plasminogen/plasmin system of cell membranes. In parallel and with regulatory and antagonistic action, four tissue inhibitors of MMPs (TIMPs) were described: TIMP-1, 2, 3, and 4. Hyperexpression of TIMP-1, 2 and 3 normally accompanies the course of tumor growth [2].

**Figure 1:**
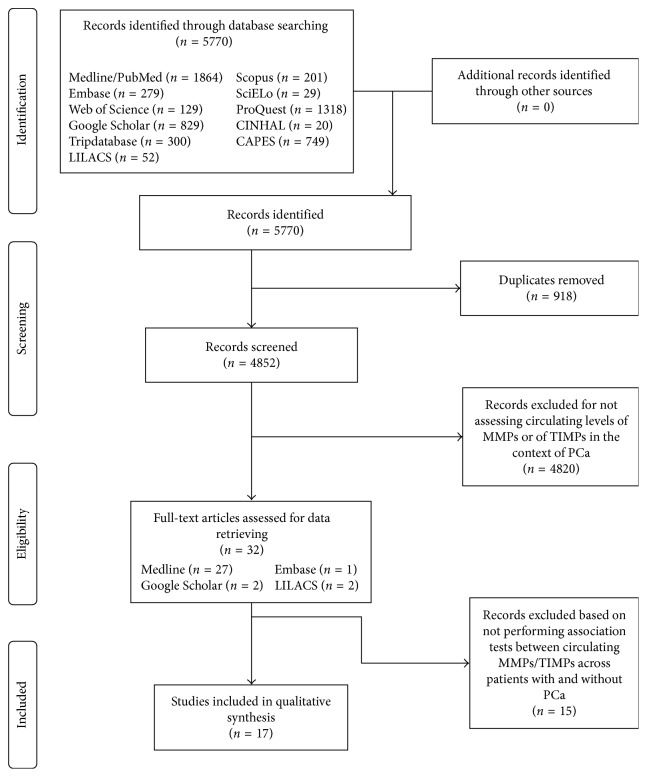
Rationale of the selection of articles.

Studies performed so far on MMPs/TIMPs to assess risk for PCa seem to yield inconclusive results, with data on specificity and sensitivity being scarce. In this context, this minireview aimed at identifying studies that correlated circulating MMPs and TIMPs with PCa, focusing on reports that aimed at having them tested as serum/plasma biomarkers and describing accuracy scores, when available. A bibliographic survey was carried out in February and March 2017, using the following key words: metalloproteinases OR inhibitors of metalloproteinases OR MMPs OR TIMPs AND prostate cancer. The following quantitative studies were identified in the following primary databases: CINAHL, 20; EMBASE, 141; Google Scholar, 500; Library COCHRANE, 0; LILACS, 52; MEDLINE, 1859; SCOPUS, 201; and Web of Science, 129, and also in the following secondary sources of information: CAPES theses and dissertations database, 749; SCIELO, 29; PROQUEST, 1318; and Tripdatabase, 295. This search, after excluding replicates, produced a total of 17 reports addressing association of plasma/serum MMPs and/or TIMPs with PCa (Figure 1), which were obtained, analyzed, and systematized as depicted in Table 1.

There is a higher prevalence of studies on MMP-2 and MMP-9. In 1998, Gohji et al. [20] accumulated evidence of the correlation between the higher serum levels of MMP-2 and tumor extension. The authors measured MMP-2 by ELISA in the serum of 98 PCa patients, with 76 BPH carriers and 70 healthy men. Serum levels of MMP-2 were significantly higher in the PCa group than in the healthy and BPH counterparts and even higher in patients with metastatic PCa. In line, Kanoh et al. [19] measured by ELISA serum MMP-2 and PSA levels of 51 PCa patients and of 39 BPH carriers. The result consisted of increasing serum levels of both along with disease progression. Very high values of MMP-2 (>950 ng/ml) and PSA (>300 ng/ml) were observed when bone metastases was observed. Those authors advocate that MMP-2 can be coupled to PSA for prognostic purposes in PCa.

In this same sense, the study by Morgia et al. [11] investigated the use of MMPs as circulating biomarkers for the diagnosis and prognosis of PCa. Levels of MMP-2, 9, and 13 were significantly higher among PCa patients than in healthy or HPB subjects. The authors concluded that serum MMPs can be used as adjuvant biomarkers (combined with PSA) for the diagnosis (MMP-13) and prognosis (MMP-2 and MMP-9) of PCa. In addition, Prior et al. [13] also measured MMP-2 (and others, including PSA) in serum (and urine) of 113 men, stating that MMP-2 assessed in combination with PSA increases sensitivity for the diagnosis of PCa.

Likewise, Zhang et al. [17] investigated enzyme activity by zymography of MMPs-2, among others, in the serum of healthy men (*n*=20), with BPH (*n*=26), with localized PCa (*n*=10), and with metastatic PCa (*n*=15). The results indicated significant differences in enzyme activity between groups for MMP-9 but not for MMP-2. Thus, unlike previous studies, it was concluded that only serum levels of MMP-9 would be correlated with the presence of malignancy and metastases.

Incorvaia et al. [9] measured serum MMP-2 and 9 in patients with breast and prostate cancer, with and without bone metastases. Regarding PCa, both MMPs were significantly higher in patients with PCa compared to control subjects, but being indistinguishable between subjects with and without bone metastases, conversely to Kanoh et al. Therefore, it was concluded that MMPs (mainly MMP-2) display low accuracy for the diagnosis of bone metastatic PCa. Salminem et al. [15] obtained the same conclusions as Incorvaia et al. [9] on the accuracy of MMP-2 and 9 in the diagnosis of bone metastatic PCa, compared to the accuracy of PSA and alkaline phosphatase, contraindicating the testing of these MMPs for diagnostic purpose. Likewise, MMP-9 was the target of Gil-Ugarteburu et al. [18], which correlated MMP-9 plasma concentrations of 235 patients (measured by ELISA) with the 1562C/T polymorphism of the promoter region of the gene. Among the findings, the authors did not identify differences in the circulating concentrations of MMP-9 in the derived subgroups or any correlation with the polymorphism investigated.

In contrast, Castellano et al. [6] evidenced that serum levels of MMP-9 and its activator, osteopontin, declined significantly 6 months after prostatectomy. They also identified a correlation between serum MMP-9 and PSA and Gleason staging values. De Cicco et al. [7] quantified MMP-2, MMP-9, TIMP-1, and TIMP-2 among other molecules in the plasma of 162 men diagnosed with PCa, having found only a significant association between low MMP-2 values (less than 206 ng/ml) and an worsened disease progression (corrected HR = 1.7 and CI = 95%).

González Rodrigues et al. [14] found unsatisfactory results when serum MMP-9 was determined by ELISA in 100 patients with indication for prostate biopsy (prospective cohort study). Of these, 32 were diagnosed with PCa with 52% classified with Gleason greater than or equal to 7. No significant difference in MMP-9 levels was found between groups with PCa and benign or uncertain histological results. No association was found between MMP-9 levels and PSA or Gleason scores.

Concerning other varieties of MMPs, Jung et al. in 1997 [10] performed ELISA assessments for plasma MMP-1, 3, and TIMP-1 on 19 nonmetastatic PCa, 18 metastatic, and 29 HPB patients, along with 35 healthy men. No difference was found in the MMP-1 means across groups. The mean concentration of MMP-3 and TIMP-1 in metastatic patients was significantly higher than in the other groups, with 10 out of the 18 metastatic cases displaying remarkably high levels of TIMP-1. They concluded that TIMP-1 can be correlated with the PCa condition. Previously, Baker et al. [4] also found higher levels of TIMP-1 (but not TIMP-2) in patients with PCa.

Serum MMP-7 was investigated by Szarvas et al. [16] using ELISA in 93 patients with focal PCa at the preoperative stage, along with 13 patients with bone metastases and 19 normal individuals. No statistically significant difference was found between PCa carriers and normal individuals. However, MMP-7 levels were significantly elevated in patients with metastatic PCa compared to focal counterparts, with specificity and sensitivity of 69 and 92%, respectively, when a cutoff point of 3.7 ng/ml was adopted.

Plasma TIMP-1 was also the subject of Oh et al. [12] in a cohort study with mean follow-up of 6.6 years. Based on 362 samples from hormone-resistant and castrated patients with metastatic PCa, patients with higher levels of plasma TIMP-1 had the lowest survival (19 versus 43 months). Values of PSA, alkaline phosphatase, and Gleason scores were also considered. Plasma TIMP-1 was shown as the best predictor of survival in patients with these characteristics and independently of other classic markers.

Bonaldi et al. [5] correlated serum levels of e-cadherin and MMP-13 on PCa patients with serum levels of total PSA, free PSA, total testosterone, and clinical evolution, measured before onset of treatment as well as three and six months afterwards. The same was done in a parallel control group. At baseline, e-cadherin titers were lower in the PCa group than in the control group while for MMP-13, differences were not noticed. With treatment, authors identified only positive correlation between PSA and e-cadherin levels in the third month of treatment. Gong et al. [8] compared circulating TIMP-1 in hormone-resistant PCa patients who underwent orchiectomy with patients responsive to hormone therapy. In the first group, plasma TIMP-1 was significantly higher.

Thus, with regard to MMPs as circulating biomarkers to diagnose and monitor PCa, we conclude that very few studies were conducted in this matter, having rendered contradictory and inconclusive data. Nonetheless, the premise of differential levels in circulating MMPs among PCa patients for a diagnostic purpose seems worth investigating in light of evidence already existent for other neoplastic entities [21], with emphasis on what concerns MMP-2, 7, and 9 and TIMP-1 in the opinion of the authors of this minireview.

Although having reviewed seventeen scientific papers, it was not possible to meta-analyze results due to methodological heterogeneities and poor description of central tendency scores. Only five articles reported mean values for the plasma/serum markers assessed, three of which for MMP-2 and MMP-9 while the others for MMP-7 and TIMP-1 each. Specificity and sensitivity were only described in 1 study [13].

Considering that the current screening diagnosis, based on serum PSA dosage and rectal examination, has a limited accuracy (mainly specificity) for differentiation of PCa from other prostatic diseases and considering the fragility of the results pointed out in this review, more studies with the aim of confirming (or excluding) MMPs and TIMPs as elective biomarkers for PCa should be welcomed, either for diagnosis, prognosis, or therapeutic referral.

## Figures and Tables

**Table 1:** Summary of the 17 articles revised.

Authors and title	Objective of the study	Design	Material and sample	Analysis method	Conclusion
Baker et al. [4]	To measure serum levels of collagenases, stromelysins, and TIMP-1 and 2 in patients with PCa, before treatment and 6 and 12 months after starting.	Prospective cohort	Test: serum of 19 individuals with metastatic PCa and 16 with PCa without metastases.	ELISA	Increase of collagenases and TIMP-1 in patients with metastatic PCa compared to those without metastases and in the former in relation to the control group with or without rheumatoid arthritis.
			Control: 21 patients with rheumatoid arthritis and 57 healthy subjects without rheumatoid arthritis.		Reduction of TIMP-1 and collagenase levels 6 months after treatment. After 12 months, the levels of collagenases remained low; however, those of TIMP-1 returned to pretreatment values.

Bonaldi et al. [5]	To dose e-cadherin and MMP-13 at the diagnosis of PCa and three and six months after treatment, comparing with the control group.	Prospective cohort	Test: plasma (EDTA) of 29 PCa patients.	ELISA	No difference between mean MMP-13 values among test and control groups at any test period.
			Control: 10 healthy men with PSA <1.5 ng/ml.		

Castellano et al. [6]	To compare levels of osteopontin (OPN), MMP-2, MMP-9, and TIMP-1.	Cross-sectional	Test: plasma (heparin) of 96 patients with PCa.	ELISA	Differences of MMP-9 and TIMP-1 (but not MMP-2) between groups; significant increase of MMP-9 and reduction of TIMP-1 in the CaP group relative to the healthy and BPH control; decreased serum levels of MMP-9 six months after radical prostatectomy.
			Control: 92 individuals with BPH and 125 healthy subjects.		

Cicco et al. [7]	Correlate preoperative serum levels of 6 markers (including MMPs-2 and 9 and TIMPs-1 and 2) with tumor staging, Gleason score, and disease-free survival.	Cross-sectional	Serum of 162 PCa carriers for MMP-2 and 9 and plasma (EDTA) for TIMP-1 and 2.	ELISA	Patients with serum levels of MMP-2 < 206 ng/ml had a higher risk of PCa progression.

Gong et al. [8]	To compare TIMP-1 levels of castrated metastatic PCa patients with noncastrated (responsive to androgen ablation therapy).	Descriptive	Test: serum of 39 castrated metastatic PCa patients.	ELISA	Higher TIMP-1 serum levels in castrated PCa patients.
			Control: 24 noncastrated metastatic PCa patients.		

Incorvaia et al. [9]	To compare levels of MMP-2 and 9 in individuals with PCa with bone metastases in relation to healthy individuals.	Cohort	Test: plasma (EDTA) of 35 patients with breast cancer and 44 with PCa with bone metastases.	ELISA	MMP-2 and MMP-9 significantly higher in PCa patients with bone metastases than in the control group.
			Control: 57 healthy patients.		

Jung et al. [10]	To compare levels of MMP-1, MMP-3, and TIMP-1 as well as the MMP-1/TIMP-1 ratio of subjects with metastatic PCa and with nonmetastatic PCa.	Cross-sectional	Plasma (heparin) of 47 patients with prostate cancer, 29 with no metastasis (T2, 3pN0M0), and 18 with metastasis (T2, 3, 4pN1, 2M1).	ELISA	Mean MMP-1 and TIMP-1 scores were significantly higher in the metastatic PCa group than in the nonmetastatic PCa, BPH, and healthy subjects groups.
			Control: 35 healthy subjects and 29 with BPH.		10 of the 18 patients with metastatic PCa presented high levels of TIMP-1.
Morgia et al. [11]	To measure plasma levels of MMPs-2, 9, and 13 of TIMP-1, and of the enzymatic activity of MMPs-2 and 9 in patients with metastatic PCa, nonmetastatic PCa, BPH, and healthy, at diagnosis and 90 days after starting treatment.	Cohort	Plasma (heparin) of 40 patients with prostate cancer, 20 with no metastasis and 20 with metastasis.	ELISA	Plasma levels of MMP-2, 9, and 13 higher at diagnosis in the PCa group with metastasis than in the other groups, with reduction after treatment.
			Control: 20 healthy patients and 20 with BPH.		Decreased TIMP-1 in the PCa group with metastasis in relation to the healthy group but without significant difference between groups.

Oh et al. [12]	To evaluate TIMP-1 as a predictor of survival in castrated PCa patients.	Survival study	Test: plasma (EDTA) of 362 castrated PCa patients; sample was divided into two groups: one with 60 (pilot group) individuals with a follow-up time of 5.8 years and the other with 302 (primary group) participants followed by 6.6 years.	ELISA	Lower survival rates among individuals with higher levels of TIMP-1 in both groups.

Prior et al. [13]	To determine sensitivity, specificity, and predictive values for MMP-2 as a biomarker for PCa.	Diagnostic study	Test: serum of 34 PCa patients.	ELISA	Increased levels of MMP-2 among subjects with PCa compared to the group without PCa.
					Sensitivity: 24.1%; specificity: 78.6%; PPV: 31.8%; NPV: 71.4%.
			Control: 79 patients without PCa.		Cutoff of 718.36 ng/ml (mean level of MMP-2 in those without PCa).

González Rodríguez et al. [14]	To dose MMP-9 in patients who underwent prostate biopsy.	Cross-sectional	Test: serum of 32 patients with positive biopsy (PCa group).	ELISA	No difference in MMP-9 levels between groups.
			Control: 58 patients with negative biopsy.		

Salminen et al. [15]	To evaluate the prognostic value of MMP-2 and MMP-9 in PCa with and without bone metastasis, comparing with ALP and PSA.	Cross-sectional and prognostic	Test: serum of 35 individuals with PCa with bone metastasis.	ELISA	No differences in MMP-2 and 9 levels between groups.
			Control: 49 individuals with PCa without bone metastasis.		MMP-2 and 9 presented low accuracy for the diagnosis of bone metastasis in PCa and were not associated with survival.

Szarvas et al. [16]	To compare serum levels of MMP-7 in PCa patients with and without metastasis and to assess its prognostic value.	Cross-sectional and prognostic	Test: serum of 93 individuals with localized PCa and 13 PCa cases with bone metastasis.	ELISA	Higher serum levels of MMP-7 in PCa patients with distant metastasis; specificity of 69% and sensitivity of 92% for detection of metastasis.
			Control: 19 healthy individuals.		

Zhang et al. [17]	To search mRNA and enzymatic activity of MMP-2 and 9 in prostatic tissue and serum of PCa patients (with and without metastasis) comparing with BPH and healthy group.	Cross-sectional	Test: serum of 15 PCa patients with metastasis and 10 without metastasis.	RT-PCR and zymography	Increased expression and enzymatic activity of MMP-9 compared to the other groups.
			Control: 26 BPH patients and 20 healthy.		
Gil-Ugarteburu et al. [18]	To correlate the 1562C/T polymorphism of the MMP-9 gene with its plasma levels.	Prospective cohort	Test: plasma (heparin) of 90 patients submitted to prostatic biopsy with positive results for PCa.	ELISA	No correlation between the gene polymorphism and plasma concentration of MMP-9.
			Control: 135 with negative biopsy for PCa.		

Kanoh et al. [19]	To correlate the serum levels of MMP-2 and PSA with the different stages of PCa.	Cross-sectional	Test: serum of 51 PCa patients.	ELISA	MMP-2 and PSA levels associated with metastatic PCa; higher levels of MMP-2 (>950 ng/ml) and PSA (>300 ng/ml) in PCa with bone metastasis.
			Control: serum of 39 BPH.		

Gohji et al. [20]	To compare MMP-2 levels between individuals with and without PCa.	Cross-sectional	Test: serum of 98 individuals with PCa without previous treatment.	ELISA	Higher levels of MMP-2 in the PCa than in the control group.
			Control: serum of 76 individuals with BPH and 70 healthy.		

BPH = benign prostate hyperplasia; MMP = matrix metalloproteinase; NPV = negative predictive value; PCa = prostate cancer; PPV = positive predictive value; TIMP = tissue inhibitor of metalloproteinase.
